# Supplementary material for: Polyketide synthase gene clusters in the ericoid mycorrhizal fungus Oidiodendron maius: genome-wide prediction, expression profiling, and functional characterization of knockout mutants
Source: Front Plant Sci. 2026 Sep 16;17:1924430. doi: 10.3389/fpls.2026.1924430 (PMC13623903; doi:10.3389/fpls.2026.1924430)
Supplement: Supplementary file 1 [file SupplementaryFile1.zip › Supplementary Materials/Supplementary Folder 1.zip!/Oidma1_AssemblyScaffolds_Repeatmasked/knownclusterblast/cluster12/jgi.p_Oidma1_116374_mibig_hits.html]

| MIBiG Protein | Description | MIBiG Cluster | MiBiG Product | % ID | % Coverage | BLAST Score | E-value |
| --- | --- | --- | --- | --- | --- | --- | --- |
| BAK26559.1 | cytochrome\_P450 | BGC0000977 | Cyclopiazonic\_acid\_biosynthetic\_gene\_cluster | 29 | 88.2828282828 | 138 | 2.9e-32 |
| BAV32149.1 | cytochrome\_P450\_monooxygenase | BGC0001373 | Sordarin\_biosynthetic\_gene\_cluster | 26 | 92.5252525253 | 129 | 1e-29 |
| ACH72911.1 | AflV | BGC0000011 | Aflatoxin/sterigmatocystin\_biosynthetic\_gene\_cluster | 24 | 96.9696969697 | 113 | 1e-24 |
| BAJ09784.1 | P450 | BGC0000146 | Solanapyrone\_biosynthetic\_gene\_cluster | 24 | 91.3131313131 | 111 | 3.8e-24 |
| AGA37280.1 | P450\_monooxygenase | BGC0000819 | Paraherquamide\_biosynthetic\_gene\_cluster | 27 | 95.5555555556 | 108 | 3.2e-23 |
| CDM36724.1 | Cytochrome\_P450 | BGC0001360 | Mycophenolic\_acid\_biosynthetic\_gene\_cluster | 37 | 33.5353535354 | 97 | 5.7e-20 |
| BAI44339.1 | P450 | BGC0000677 | Cyclooctatin\_biosynthetic\_gene\_cluster | 37 | 35.9595959596 | 88 | 2e-17 |
| AAC49192.1 | putative\_p450\_monooxygenase | BGC0000152 | Sterigmatocystin\_biosynthetic\_gene\_cluster | 31 | 31.5151515152 | 77 | 6.1e-14 |
| ADY00131.1 | putative\_P450\_monooxygenase | BGC0000104 | Mycophenolic\_acid\_biosynthetic\_gene\_cluster | 41 | 25.2525252525 | 76 | 1.4e-13 |
| AAS90045.1 | CypA | BGC0000009 | Aflatoxin\_biosynthetic\_gene\_cluster | 25 | 73.7373737374 | 71 | 4.4e-12 |
